# Supplementary material for: The DBB Family in Populus trichocarpa: Identification, Characterization, Evolution and Expression Profiles
Source: Molecules. 2024 Apr 17;29(8):1823. doi: 10.3390/molecules29081823 (PMC11054233; doi:10.3390/molecules29081823)
Supplement: Supplementary file 1 [file molecules-29-01823-s001.zip › Table S1.pdf]

Table S1. Detailed information about *ZmGLK* genes in maize.

Gene name	Sequences ID	MW(Da)	PI	CDS Length(bp)	Size(aa)
Populus	Potri.001G384000.1	31852.75	5.75	894	297
trichocarp	Potri.002G028200.1	34336.37	5.79	933	310
	Potri.004G161000.1	42102.84	5.97	621	378
	Potri.004G162600.3	88462.73	5.75	612	791
	Potri.005G117100.2	20506.21	7.05	555	184
	Potri.005G234500.1	34288.43	6.20	936	311
	Potri.007G015200.2	20515.17	6.23	558	185
	Potri.007G130100.2	26022.39	4.80	708	235
	Potri.009G122000.1	24021.86	6.39	654	217
	Potri.009G124400.1	66923.85	7.44	612	596
	Potri.011G105400.1	63262.02	6.07	897	579
	Potri.017G028300.1	25989.39	4.77	717	238
Arabidopsis	AT1G06040.1	27641.03	5.42	747	248
thaliana	AT1G06040.2	19718.44	6.06	534	177
	AT1G75540.1	35533.61	6.44	996	331
	AT1G78600.1	32863.55	5.89	900	299
	AT1G78600.2	35079.29	6.11	960	319
	AT2G21320.1	18891.01	5.71	519	172
	AT2G24790.2	24305.41	5.23	663	220
	AT2G31380.1	26656.92	4.90	717	238
	AT2G47890.2	26130.62	4.96	714	237
	AT4G10240.1	17858.96	4.98	489	162
	AT4G38960.1	20146.62	5.36	552	183
	AT4G38960.3	25116.58	5.91	681	226
	AT4G39070.1	26779.66	6.04	729	242
	AT5G15840.2	30601.50	5.78	825	274
Oryza sativa	LOC_Os01g10580.1	37090.17	5.93	1074	357
	LOC_Os02g39360.1	29148.43	4.93	816	271
	LOC_Os02g43170.1	28235.40	5.36	810	269
	LOC_Os02g43170.2	21619.04	5.18	627	208
	LOC_Os04g41560.2	27136.68	4.89	774	257
	LOC_Os04g41560.4	18937.53	5.47	546	181
	LOC_Os04g45690.1	26733.80	5.12	753	250
	LOC_Os05g11510.1	39399.78	5.48	1137	378
	LOC_Os06g05890.1	38395.67	5.26	1083	360
	LOC_Os06g49880.1	31914.66	5.08	927	308
	LOC_Os06g49880.2	31786.53	5.08	924	307
	LOC_Os09g35880.1	23366.18	5.90	636	211
	LOC_Os12g10660.1	21629.79	4.99	633	210
Zea mays	GRMZM2G018876_P01	27665.26	4.87	780	259
	GRMZM2G021777_P02	30470.81	4.54	903	300

	GRMZM2G028594_P01	40113.65	5.07	1128	375
	GRMZM2G028594_P03	46942.63	5.29	1308	435
	GRMZM2G070446_P01	40436.89	5.08	1128	375
	GRMZM2G075562_P02	25112.18	5.26	699	232
	GRMZM2G095299_P01	27476.02	5.82	765	254
	GRMZM2G098442_P01	27913.67	4.80	771	256
	GRMZM2G110541_P01	15378.82	9.56	429	142
	GRMZM2G118884_P01	31873.54	4.73	927	308
	GRMZM2G131982_P02	27823.72	5.19	777	258
	GRMZM2G143718_P01	22716.35	5.42	621	206
	GRMZM2G143718_P02	20617.18	5.50	558	185
	GRMZM2G422644_P01	22545.25	5.54	618	205
	GRMZM2G422644_P02	17737.94	9.21	471	156
	GRMZM5G834596_P01	36806.01	5.30	1059	352
	GRMZM5G834596_P03	36806.01	5.30	1059	352
	GRMZM5G834596_P05	36806.01	5.30	1059	352
	GRMZM5G834596_P06	36806.01	5.30	1059	352
Physcomitrella	Pp3c11_20560	24828.00	6.20	666	221
patens	Pp3c14_10320	36188.00	7.02	1017	338
	Pp3c17_14230	41253.64	6.79	1155	384
	Pp3c17_6790	42815.99	5.85	1620	387
	Pp3c7_25600	33423.11	5.28	1095	306
	Pp3c7_5280	24551.67	6.26	663	220
Selaginella	102790	10339.91	6.15	288	96
moellendorffii	111647	12151.10	6.86	336	111
	271535	30613.49	5.05	858	285
	444777	38352.35	8.50	1071	356
Picea abies	MA_10433513g0010	19287.64	7.10	498	174
	MA_128658g0010	32059.09	5.93	996	293
Amborella	scaffold00001.223	27245.76	6.40	738	245
trichopoda	scaffold00099.135	20837.29	6.21	561	186
	scaffold00182.23	25484.70	6.50	684	227
