# Supplementary material for: The DBB Family in Populus trichocarpa: Identification, Characterization, Evolution and Expression Profiles
Source: Molecules. 2024 Apr 17;29(8):1823. doi: 10.3390/molecules29081823 (PMC11054233; doi:10.3390/molecules29081823)
Supplement: Supplementary file 1 [file molecules-29-01823-s001.zip › Table S2.pdf]

Table S2. The MEME motif sequences and lengths of PtrDBB proteins in *Populus trichocarp*.

Motif	Width	Best possible match
1	50	MKIQCDVCEKAEATVFCCADEAALCDACDEKVHAANKLASKHQRVSLAHP
2	36	PKCDICQEAPAFFFCZEDRALLCRECDMPIHTAGEH
3	29	THNRFLLTGVKVELPSSKPGASSSSGQSP
4	50	ETRRGQNQPPKLTARENRQNRRASPVPMVENNSDGDGKVDKKLIDLNAKP
5	15	STIPGWHVDDFLDPS
6	40	NQQYGYPDLGGQKGDKGKKGDSDCKANRRLADDEFDDPZC
7	50	WLADMGLFGEQLPQEALAAAEVPQLPISPPTNVNACRPPKFSMPHKKPRI
8	41	TTPFGFCKIDDGLLPFMDAHDLERNMSSFSSESLGLWVPQA
9	46	APVNSNPPAVPSTLSANTEINKGGDNLVTNEGFGSTTSSTISEYLM
10	50	GEKKTLETKSCPVSRRGTLLPLANPCNQVFPVNVCGVGEFGPAKLPYSGG
