# Supplementary material for: The DBB Family in Populus trichocarpa: Identification, Characterization, Evolution and Expression Profiles
Source: Molecules. 2024 Apr 17;29(8):1823. doi: 10.3390/molecules29081823 (PMC11054233; doi:10.3390/molecules29081823)
Supplement: Supplementary file 1 [file molecules-29-01823-s001.zip › Table S4.pdf]

Table S4. The primers of qRT-PCR of 12 *PtrDBBs* and *Ptr18S*.

**Genes**		**Primers used in qRT-PCR(5′→3′)**
PtrDBB1	Forwards	GATAGTGGCAAACACGGGGA
	Reverse	GAGCTATCTGGCACCTGACC
PtrDBB2	Forwards	ACGAGCATACCCAGAAGCAC
	Reverse	CAGGAACGAGATCACCACCA
PtrDBB3	Forwards	GACAGCATCAGCAACAACGG
	Reverse	AAAATCATCGATGCGCCAGC
PtrDBB4	Forwards	CCTGCGACGAGAAGGTTCAT
	Reverse	GCAGAGCGAACTGCCATCTA
PtrDBB5	Forwards	ACCCTTTGTGACGCTTGTGA
	Reverse	TTCACTGGGATTCGCCAGAC
PtrDBB6	Forwards	ACAGCAAACGAGCATACCCA
	Reverse	GCCACTACTGGTAACGGAGG
PtrDBB7	Forwards	CTGATGAGGCTGCACTTTGC
	Reverse	TCACTGGGATTTGCCAGACC
PtrDBB8	Forwards	GGAAATTGGTGCGCATGGAG
	Reverse	TGGCTCAGTCTCAGTTGCAG
PtrDBB9	Forwards	TTTCTGGCCACTGGAATCCG
	Reverse	AAAACTCGATGCATGCTGCC
PtrDBB10	Forwards	AGCTGATGAAGCCGCTCTTT
	Reverse	CTGTGGAACAGCGCTAGGAT
PtrDBB11	Forwards	GGCAGTTCCCCAAATCCCTT
	Reverse	TGCCACGCTGTTCACTGTAA
PtrDBB12	Forwards	TCGAGTTGCTCCAAGGACAC
	Reverse	AGCAAGTCATCAACTGCCCA
Ptr18S	Forwards	AAAATCATTGTAGGCCATTGTCG
	Reverse	ACTAAATTAAGCCAGCGGGAGTG
