# Supplementary material for: The DBB Family in Populus trichocarpa: Identification, Characterization, Evolution and Expression Profiles
Source: Molecules. 2024 Apr 17;29(8):1823. doi: 10.3390/molecules29081823 (PMC11054233; doi:10.3390/molecules29081823)
Supplement: Supplementary file 1 [file molecules-29-01823-s001.zip › Table S7.pdf]

Table S6.

(A) Expression level of *PtrDBB* genes in response to light/dark treatments.

Time / Gene ID	light	dark
PtrDBB1	1	1
PtrDBB2	1.73	1
PtrDBB3	8.32	1
PtrDBB4	2.6	1
PtrDBB5	7.72	1
PtrDBB6	1.56	1
PtrDBB7	3.21	1
PtrDBB8	2.15	1
PtrDBB9	10.35	1
PtrDBB10	2.55	1
PtrDBB11	2.8	1
PtrDBB12	1.89	1

(B) Expression level of *PtrDBB* genes in response to drought stress.

Time / Gene ID	0h	1h	3h	6h	12h	24h
PtrDBB1	1	1.62	1.68	2.79	2.55	2.83
PtrDBB2	1	1.53	3.58	2.21	2.32	3.11
PtrDBB3	1	1	6.71	8.55	15.09	5.32
PtrDBB4	1	3.68	3.62	4.78	2.67	3.92
PtrDBB5	1	2.91	5.78	8.82	10.67	6.56
PtrDBB6	1	1	4.77	7.32	12.19	8.29
PtrDBB7	1	3.77	4.32	8.19	13.29	5.51
PtrDBB8	1	3.53	2.99	2.82	4.85	3.58
PtrDBB9	1	1.53	3.99	7.82	14.81	6.58
PtrDBB10	1	0.62	0.55	0.58	0.42	0.47
PtrDBB11	1	1.94	3.32	2.11	2.78	4.72
PtrDBB12	1	4.91	2.78	3.82	3.91	3.56

(C) Expression level of *PtrDBB* genes in response to ABA treatment.

Time / Gene ID	0h	1h	3h	6h	12h	24h
PtrDBB1	1	0.62	0.68	3.79	2.55	2.83
PtrDBB2	1	2.68	3.71	3.55	4.09	4.32
PtrDBB3	1	1	2.71	3.55	4.09	4.32
PtrDBB4	1	4.11	6.62	8.78	8.67	5.92
PtrDBB5	1	2.91	5.78	8.82	9.91	6.56
PtrDBB6	1	2.8	1.6	2.34	3.39	3.59
PtrDBB7	1	0.82	1.73	2.31	2.02	1.58
PtrDBB8	1	1.77	3.32	3.11	2.29	3.51

PtrDBB9	1	3.53	3.99	7.82	14.85	6.58
PtrDBB10	1	2.91	5.78	8.82	12.91	8.56
PtrDBB11	1	2.46	6.32	10.11	12.29	3.51
PtrDBB12	1	4.91	5.78	6.82	8.91	5.56

(D) Expression level of *PtrDBB* genes in response to MeJA treatment.

**Time / Gene ID**	**0h**	**1h**	**3h**	**6h**	**12h**	**24h**
PtrDBB1	1	2.12	2.98	3.55	3.18	3.33
PtrDBB2	1	2.89	1.56	3.37	4.83	2.67
PtrDBB3	1	2.56	3.86	4.59	4.57	3.85
PtrDBB4	1	0.79	0.83	1.46	1.83	2.22
PtrDBB5	1	4.67	4.22	3.69	3.22	4.81
PtrDBB6	1	6.85	8.55	13.41	7.22	5.31
PtrDBB7	1	3.54	4.66	7.62	7.81	4.63
PtrDBB8	1	1.89	2.33	2.51	3.31	3.78
PtrDBB9	1	5.16	5.42	8.89	16.36	8.92
PtrDBB10	1	0.95	2.59	3.15	3.59	4.26
PtrDBB11	1	1	10.89	18.33	16.51	6.34
PtrDBB12	1	0.67	0.89	0.73	0.79	0.56
