# Supplementary figures and images for: The DBB Family in Populus trichocarpa: Identification, Characterization, Evolution and Expression Profiles
Source: Molecules. 2024 Apr 17;29(8):1823. doi: 10.3390/molecules29081823 (PMC11054233; doi:10.3390/molecules29081823)

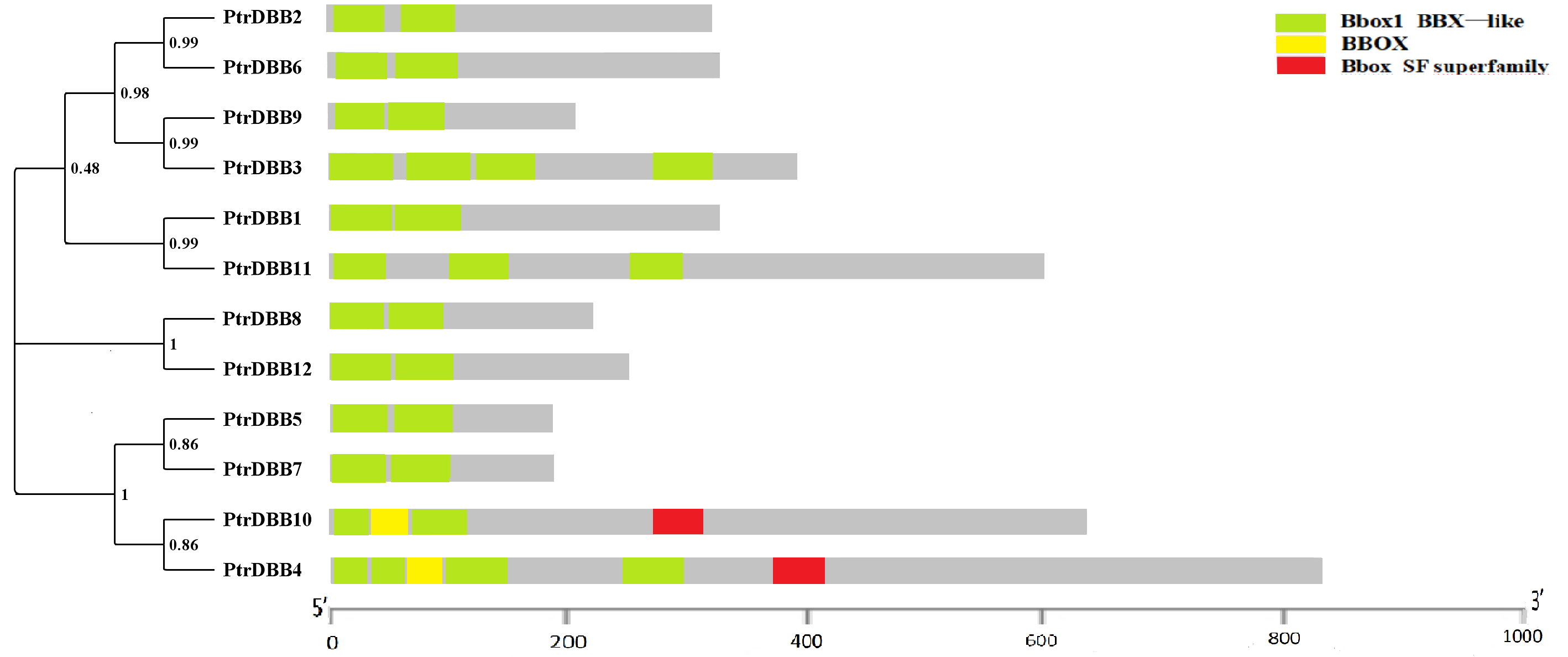

Supplement: Supplementary file 1 [file molecules-29-01823-s001.zip › Figure S1.png]
